# Information Adequacy in Histopathology Request Forms: A Milestone in Making a Communication Bridge Between Confusion and Clarity in Medical Diagnosis

**DOI:** 10.5146/tjpath.2022.01595

**Published:** 2023-09-15

**Authors:** Fariba Abbasi, Yasaman Asghari, Zahra Niazkhani

**Affiliations:** Solid Tumor Research Center, Cellular and Molecular Medicine Research Institute, Urmia University of Medical Sciences, Urmia, Iran; Department of Pathology, School of Medicine, Urmia University of Medical Sciences, Urmia, Iran; Student Research Committee, Urmia University of Medical Sciences, Urmia, Iran; Nephrology and Kidney Transplant Research Center, Clinical Research Institute, Urmia University of Medical Sciences, Urmia, Iran; Health Care Governance, Erasmus School of Health Policy & Management, Erasmus University Rotterdam, Rotterdam, The Netherlands

**Keywords:** Information adequacy, Clinical information, Patient safety, Medical errors, Request form

## Abstract

***Objective:*** Information contained in request forms for histopathological examinations plays a critical role in the microscopic interpretation of tissue changes. Despite its importance, studies have shown inadequacies in the information communicated by clinicians. This study aimed to determine how well the necessary information is provided on the histopathology request forms and to compare its variability among different departments of a hospital.

***Material and Methods:*** A retrospective, 3-month, cross-sectional study was conducted to evaluate all consecutive histopathology request forms received from different departments of a tertiary, academic hospital for three months, regarding the documentation of 12 criteria.

***Results:*** None of the 2040 requests received had all the required items. Four items of specimen description, laboratory and imaging findings, and physician contact number were available only in less than 12.5% (range between 0.05 to 12.45%) of the requests. However, four other items of patient name and contact number, physician name, and anatomical site of the lesion were documented in more than 90%. The median number of the documented items was the highest in the surgery and orthopedics (9 items) and the lowest in the pulmonology department (7 items). Comparison between departments showed that the documentation of items in the surgery department were significantly better than that of the ENT, urology, and internal medicine departments (p<0.001). Also, the internal medicine department was significantly different from all other departments (p<0.001) except neurosurgery (p=0.88).

***Conclusion:*** Our results point out a serious gap in the adequacy of pathology request forms, especially clinical items. Given the implication of such information to ensure patient safety, further studies are recommended to evaluate the impact of educational and supportive computerized interventions such as clinician education and barcoding and specimen tracking systems to help fill in the required items completely.

## INTRODUCTION

Nowadays, histopathological examination of specimens is not only an auxiliary means of diagnosis, but also a useful method to assist clinicians with appropriate therapy, proper prognosis prediction, and critical decisions in patient management (1–3). In the past, the quality of laboratory examinations was determined by the accuracy of the analytical phase (4). Following the improvements in the analytical techniques, these are no longer the main cause of errors in the laboratory testing processes (4–6). Current research shows that up to 60-77% of total laboratory errors happen in the pre-analytical phase (7–9). One of the main sources of the errors in this phase is the inadequacy of the information provided on the lab request forms by requesting clinicians (2,5).

Histopathology request forms are the main means of communication between clinicians and pathologists by including patient demographics and clinical information and the details of test requests, specimens, and requesting physicians. Each of the items in the request forms has its own critical role in this communication. For example, documenting patient age and gender can help histopathologists in the differential diagnosis of lesions (3). Also, providing patients’ correct full names can help to find more information about their previous medical history or the results of other diagnostic procedures through health information systems (3). Besides these, the adequacy of especially clinical information such as clinical history and differential diagnosis on the request forms can impact the turn-around time of histopathology examinations and their on-time interpretation (10).

Despite the importance of these items, studies have documented inadequacies in the communication of such information, pointing out many errors in the completion of the request forms (3,5,6,11,12). Insufficient, inaccurate, and illegible information in these forms are only some of the associated errors. It has even been reported in a study that up to 10% of the samples received in the pathology laboratories were not accompanied by their request forms (2). Such errors happen mainly because the requesting clinicians usually overlook their primary role and underestimate their responsibility in the pre-analytical quality assurance of the diagnostic procedures (2,3,5). Errors in the request forms can result in detrimental patient and process outcomes such as the inappropriate interpretation of morphological changes in tissues and even misdiagnosis, which in turn poses serious threats to the patients’ safety, or they might lead to extra tests such as unnecessary staining and delayed test interpretation (10,12–14). They are also responsible for the waste of both money and the pathologists’ valuable time (3,12–15).

It is noteworthy to mention that histopathologists do not see patients personally. Therefore, they are greatly dependent on complete and detailed patient information on the request forms that accompany the histopathological specimens for making an accurate diagnosis (2,16). When the responsibility to care for a patient is handed off from a requesting physician to a histopathologist, any communication breakdown in transferring the required demographics and clinical information can affect the correct interpretation and definitive diagnosis (4–6). Meanwhile, if the requesting physicians continue to underestimate the importance of their role and the required items on the request forms, such errors will continue to happen. Therefore, identifying and understanding such errors and planning effective interventions to tackle them are critical. When trying to review the adequacy of data items in the histopathology requests provided by clinicians in the healthcare context and the magnitude and types of such errors, we unfortunately could not find any relevant report. Therefore, we aimed to evaluate the completeness of information on the request forms accompanying histopathology samples, and its variation among different departments, in a pathology laboratory of a tertiary care hospital.

## MATERIALS and METHODS

This was a retrospective, cross-sectional study in a 490-bed tertiary care teaching hospital in Urmia, Iran. The hospital is the main referral hospital in the region and has broad specialty and subspecialty departments. There was a standardized histopathology request form that all the departments across the hospital used to send their specimens to the pathology department. The items that were the focus of this study were available in this form for documentation. The optimal sample size for this study based on the statistical equation (with α=0.05, d=0.05, and p=0.8) was calculated to be 260 request forms per month, considering 4% drop-out. During a 3-month period between 21st of December 2017 till 21st of March 2018, we examined all consecutive pathology request forms received at the pathology laboratory of the hospital from different inpatient wards of the hospital. Hardcopies of these requests were retrieved from the archive and manually studied according to the purposes of the present study. Similar to the previous studies (2,12,16), and also the study feasibility in our setting, requests for cytological examination and also those received from the outpatient clinics of the hospital were all excluded (although we acknowledge that they are also subject to misinterpretation without clinical information). Each request form was visually assessed for the presence and completeness of the following necessary items: patients’ full name, age, gender and contact number, clinical history, provisional/differential diagnosis, anatomical site of the specimen, specimen description, relevant laboratory, imaging and endoscopic findings, and the full name and contact number of the requesting clinicians, as studied in other studies (3,14,16,17).

The study was approved by the ethics committee of the Urmia University of Medical Sciences (ethics reference number IR.UMSU.REC.1395.592) and performed according to the Helsinki Declaration. Patients’ and the requesting providers’ confidentiality was maintained.

### Statistical Analysis

We used appropriate descriptive statistics to report our results. The statistical differences were determined by the X2 test. Because some comparisons involve many small cell frequencies, leading to more than 20% of the expected frequencies to be less than 5, we also calculated Fisher’s exact test. Moreover, we used the Kruskal-Wallis test, followed by non-parametric pairwise group comparisons of post-hoc for significant results to compare departments, because the assumptions of the one-way ANOVA test were not met. A p-value of less than 0.05 was considered significant. Statistical analysis was performed using the Statistical Package for Social Sciences (SPSS, version 17).

## RESULTS

From 2040 pathology request forms, 46.7% (952) were sent from the surgery department followed by 28.3% (577) from the internal medicine and 11.2% (229) from Ear, Nose, and Throat (ENT) departments.

None of the request forms received had all the required items. The clinician contact number was the least completed one (99.95% missing), followed by imaging findings (94.80%), description of the specimens (87.94%), and laboratory findings (87.54%). Three other important clinical items of clinical history consisting of the differential/provisional diagnosis, and the anatomical site of the specimen were not mentioned in 19.96%, 17.4%, and 7.15% of cases, respectively. Even patient identifiers of gender and age were missing in 27.75% and 8.04% of cases. There were significant differences among different departments regarding the documentation of the following items: patients’ age, gender and contact number, clinical history, provisional/differential diagnosis, anatomical site of the specimen, laboratory findings (all with p<0.001), description of the specimen (with p=0.006), and clinician name (p=0.01) (Table 1). Table 1 shows the details of the department(s) from which the difference originates.

**Table 1:** Completion rates of the required items on the pathology request forms sent from different departments.

**Items assessed**	**Departments involved**						P value*		**All departments (documented items)** **N=2040** **n (%)**	**All departments (missing items)** **N=2040** **n (%)**
	**Urology** **N=196** **n (%)**	**Surgery** **N=952** **n (%)**	**Orthopedics** **N=76** **n (%)**	**Internal medicine** **N=577** **n (%)**	**ENT** **N=229** **n (%)**	**Neurosurgery** **N=10** **n (%)**				
**Patient identifiers**										
1. Name	196 (100)	951 (99.9)	76 (100)	576 (99.8)	227 (99.1)	10 (100)	0.27**		2036 (99.8)	4 (0.2)
2. Age	186 (94.9)^a^	938 (98.5)^b^	73 (96.1)^a,b,c^	482 (83.5)^c^	189 (82.5)^c^	8 (80)^a,c^	<0.001		1876 (91.96)	164 (8.04)
3. Gender	113 (57.7)^a^	788 (82.8)^b^	52 (68.4)^a^	369 (64)^a^	146 (63.8)^a^	6 (60)^a,b^	<0.001		1474 (72.25)	566 (27.75)
4. Contact number	187 (95.4)^a,b^	917 (96.3)^b^	73 (96.1)^a,b^	514 (89.1)^a^	210 (91.7)^a^	10 (100)^a,b^	<0.001		1911 (93.82)	129 (6.18)
**Clinical items**										
5. Clinical history	147 (75)a	831 (87.3)^b^	70 (92.1)^b^	366 (63.4)^c^	210 (91.7)^b^	9 (90)^a,b,c^	<0.001		1633 (80.04)	407 (19.96)
6. Provisional/differential diagnosis	169 (86.2)^a,b,c^	857 (90)c	57 (75)^b,d^	387 (67.1)^d^	208 (90.8)^a,c^	7 (70)^a,b,c,d^	<0.001		1685 (82.6)	355 (17.4)
7. Laboratory findings	35 (17.9)^a^	86 (9)^b^	11 (14.5)^a,b^	116 (20.1)^a^	4 (1.7)^c^	2 (20)^a,b^	<0.001		254 (12.45)	1786 (87.55)
8. Imaging and endoscopic findings	10 (5.1)	44 (4.6)	5 (6.6)	37 (6.4)	9 (3.9)	1 (10)	0.56		106 (5.2)	1934 (94.8)
**Specimen associated items**										
9. Anatomical site of the specimen	190 (96.9)^a,b^	913 (95.9)^a,b^	75 (98.7)^b^	492 (85.3)^c^	216 (94.3)^a,b^	8 (80)^a,c^	<0.001		1894 (92.84)	146 (7.16)
10. Description of the specimen	0 (0)^a^	35 (3.7)^a,b^	1 (1.3)^a,b^	8 (1.4)^a,b^	5 (2.2)^a,b^	1 (10)^b^	0.006**		246 (12.05)	1794 (87.95)
**Clinician identifiers**										
11. Name	195 (99.5)^a,b^	940 (98.7)^a,b^	76 (100)^a,b^	572 (99.1)^b^	220 (96.1)^a^	10 (100)^a,b^	0.01**	2013 (98.68)		27 (1.32)
12. Contact number	0 (0)	0 (0)	0 (0)	1 (0.2)	0 (0)	0 (0)	0.77**	1 (0.05)		2039 (99.95)

**ENT:** Ear, Nose, Throat. * With X^2^ test, ** With Fisher’s Exact test. Note: Each subscript letter denotes a subset of department categories whose column proportions do not differ significantly from each other at the 0.05 level

Comparing subspecialty wards of internal medicine together, there were also differences in the completion of the items such as clinical history, differential diagnosis, and anatomical site of the specimens (supplementary material). In these subspecialties, the missed clinical history of patients in the request forms varied between 9.3% of cases in the gastroenterology (GE) ward to 57% in the pulmonology ward. Imaging findings were absent in more than 90% of cases in all internal medicine wards (range between 90 to 95.65% missing). All the request forms sent from the rheumatology ward lacked any description of the specimens sent. This item was also missing in more than 97.1% of cases in all other internal medicine wards (range between 97.1-100% missing). Even, no differential diagnosis was provided in 48.1 % of the request forms sent from the oncology ward, 43.5 % from the nephrology, 39.5% from the GE, and 30% from the rheumatology wards. The anatomical site of the specimens was not available in 25.6% of the request forms of the GE, 22.11% of the oncology, and 21.73% of the nephrology wards.

**Figure 1:**
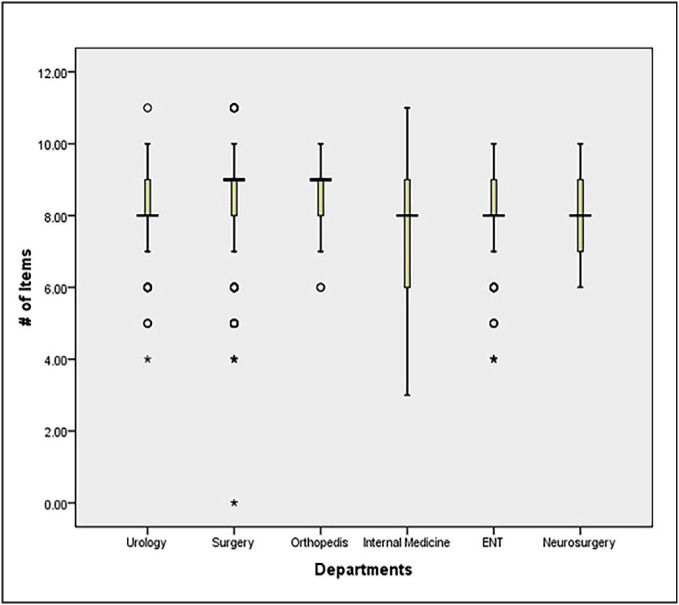
A box plot comparing the departments.

The median number of documented items (Interquartile Range (IQR)) was 9 (8-9) (minimum of 0 and maximum of 11 out of 12 required items). The median number of documented items was highest in surgery and orthopedics (9 (8-9)) and the lowest in the internal medicine department (8 (6-8)) (Table 2) (Figure 1). Among the subspecialty wards, the gastroenterology ward had the highest median number of the documented items (9 (7-9)) and the pulmonology ward the least (7 (5-8)). Comparison between departments with the post-hoc test showed that the documentation of items in the surgery department were significantly better than that of the ENT, urology, and internal medicine departments (p<0.001). Also, the internal medicine department was significantly different from all other departments (p<0.001) except neurosurgery (p=0.88). However, subspecialty wards in internal medicine department were similar to each other (p=0.06).

**Table 2:** Comparison between different departments and subspecialty wards regarding the median number of documented items on the pathology request forms.

**Ward**	**Number of request forms**	**Medians**	**Interquartile ranges**	**95% Confidence Interval**	**Minimum number of documented items**	**Maximum number of documented items**
Urology	196	8	8 - 9	8.07-8.40	4	11
Surgery *	952	9	8 - 9	8.57-8.70	0	11
Orthopedics	76	9	8 - 9	8.28-8.68	6	10
ENT	229	8	8 - 9	8.01-8.30	4	10
Neurosurgery	10	8	7 - 9.25	7.25-9.14	6	10
Internal Medicine*	577	8	6-9	7.37-7.63	3	11
General	74	8	7 - 9	7.63-8.22	5	11
Nephrology	69	8	8 - 9	8.08-8.63	5	11
Gastroenterology	43	9	7 - 9	7.94-8.75	5	11
Oncology	104	8	7 - 9	7.66-8.18	4	11
Rheumatology	50	8	7 - 9	7.49-8.22	5	10
Respiratory	237	7	5 - 8	6.50-6.92	3	10
Total	2040	9	8 - 9	8.16-8.27	0	11

* Significant difference with other departments with the post-hoc test (at p<0.001) (please see for further details in the main text)

## DISCUSSION

Our results showed that the completeness of information on the request forms in our study was suboptimal: none of the request forms contained all the required study items that were vital for the interpretation of the results and final diagnosis by pathologists. It also showed that different departments had varying request form completion rates. The gaps in the adequacy of the request forms were more notable in the internal medicine wards compared with the surgical wards. This is somehow in contrast to the literature comparing internal medicine wards with surgery, in which physicians more often completed clinical details and diagnosis than surgeons did (12). As most of our samples were from the surgery ward, it seems that the workload is not an important factor in the incomplete filling of the requests. Also, a very surprising result in our study was the availability of some items only in less than 12.5% of the requests, including the description of specimens, laboratory tests, and imaging findings. There were even gaps in the documentation of critical information such as clinical history and differential diagnosis.

Gaps in the documentation of essential data items in the request forms can profoundly impact the effectiveness of pathology tests and their contribution in proper patient diagnosis and management (18). Despite its importance, several researchers have so far documented the paucity of relevant clinical and non-clinical details being provided on laboratory specimen request forms (2,19). Similar to another study (2), we found that all the request forms had some sort of data gaps. Other studies have reported that only as low as 3% of the request forms studied had all the required information (16,20). Such low data completion rates affect the histopathology interpretations in many different ways and undermine the contribution of pathology tests to manage patients effectively (21). For example, radiology findings enhance the value of clinical data and assist in the interpretation of histopathologic findings. Similarly, the lack of clinical items limits the interpretation of the histopathology results because clinical diagnosis, clinical history, and clinical findings commonly serve as a screening method for selecting possible histopathological diagnoses. In our study, only the four items of patient name, patient phone number, anatomic location of the lesion, and the name of requesting physician were mentioned in more than 90% of the request forms. Other studies have found more than 90% completion for the four items of patient name and gender, name of requesting clinician, and date of biopsy (2,8). In another study, patient name, age and address, specimen description, and clinical history were found to have been documented in more than 90% of the cases (12). In our study, the patient name was missing in 2% of the cases. In such cases when the requesting wards are known, normally the pathology personnel immediately contact the wards to solicit the information, and if such critical information is still unknown the request is rejected. Also, patient age was recorded in only 82% of the request forms similar to 86.4% found in the study of Adegoke et al. (4). Besides its implication for accurate interpretation of the results, this missing information can lead to a big challenge for proper research and epidemiological studies, and therefore its proper completion should be emphasized.

Among all data items in the request forms, adequate clinical history and provisional/differential diagnosis are especially important, mainly because they have a critical role in the correct interpretation of the pathology results. These data help to define the need for, and the nature of, special studies that can be performed on the specimen for an accurate diagnosis. Unfortunately, clinical history and differential diagnosis were absent in 19.95 and 17.4% of the cases, respectively, in our study. In the study by Nakhle et al., with a 77% rate of errors of discrepant or missing information items, the most common deficiency was “no clinical history or diagnosis present on the requisition slip,” which represented 40% of all deficiencies (22). Other studies also reported missing rates of 50 to 85.3% for the clinical history (20,23). Although our figure is much lower than those and also the 34% of the cases with no clinical history reported by Sharif et al. (3), our rate is still much higher than those studies reporting 6.1% and 5.4% srates of missing clinical details and diagnosis, respectively (12,16). The differential diagnosis was absent in 20.4% of our request forms. Burton and Stephenson reported a missing rate of 46.9% of the forms regarding this item (12). In another study, 19.1% of the forms lacked a diagnosis, and among those with a diagnosis, only an abbreviation was provided in 37.3% (5). Such clinical notes enable pathologists to further narrow down the differential diagnosis in their interpretations and therefore should be the focus of future interventions to tackle them.

A surprising finding was the presence of the contact number of the requesting physician in merely one form out of 2040 request forms. Compared to our results, lower rates of 23% and 33.3% missing regarding this item were found in two other studies (3,12). The absence of this item can delay the pathology reporting process in cases where more clinical information is needed for pathologists; and then, trying to find the clinician’s phone number to get the information would be a time-wasting exercise. It should also be noted that the urgent results could rapidly be conveyed back to the requesting clinician if the clinician’s contact number is present on the request forms.

Our setting is a teaching hospital; then, it is natural to imagine the individuals signing the request forms are aware of the importance of filling out the requests correctly. However, unfortunately, the results we found were not very compelling. This may be due to the lack of standard and updated forms. To remedy the current situation, we recommend developing newer standard pathology request forms with the contribution of the requesting clinicians, and also to develop guidelines for the pathology reception staff in order to deal with these gaps, such as rejecting the samples without accompanying complete request forms. This will especially hold true for cases where their clinical data are vital for correct interpretation. Many other interventions can also improve the practice of providing complete sets of data to pathologists. One of the effective interventions is to plan educational sessions for clinicians and intense communication with them in order to emphasize the importance of the correct and complete filling of the request forms. A study found that after educating clinicians with various interactive media, there were significant improvements in the completion rate of required data items such as age, hospital number, clinician name, clinical diagnosis, and specimen type (24). Other effective means can be the use of computerized lab test request systems that can guide the requesting clinicians to properly send a complete pathology lab request (25). Moreover, barcoding and specimen tracking systems such as radio frequency identification (RFID)-based systems have been found useful in this regard (26,27). With the help of such systems, many required items, and especially demographics and lab and radiology findings, can automatically be filled in by available data in the patients’ electronic medical records and therefore clinicians can focus on the communication of essential clinical items on the request forms.

### Limitations

Although this study was limited to only one hospital, it is likely that the results are similar for other academic hospitals in the country. However, we recommend conducting further studies in other settings to document the magnitude of such gaps in the request forms. Another limitation of this study was our inability to assess the effect of these poorly completed request forms on proper diagnosis and patients’ management due to the retrospective nature of data collection. Due to the same reason, we were also unable to evaluate the cases individually in their own context to see whether or not there was enough information depending on the disease (e.g., clinical and radiological information) for pathologists to make a better diagnosis. This is mainly relevant because using the contact information, many contacts are made to solicit further information from patients and the requesting wards, but no information was available on such workarounds despite being very time-consuming for the pathology department. One more issue to note is that missing clinical information may yield a misdiagnosis depending on the diseases as well, so depending on the cases the requirement of the clinical information would change. For example, dermatopathology cases require detailed differential diagnosis but rarely radiologic findings. Therefore, it is necessary that the cases are evaluated individually and in their context to see whether there is enough information or not.

## CONCLUSIONS

Our results show several weaknesses and gaps in the completion of the pathology request forms in routine care. Although our study was conducted almost four years ago, no changes have unfortunately been made in the handling of the requests and samples despite complaints from pathologists and pathology department personnel, mainly because their importance and magnitude (and likely effects on correct diagnosis) are not well-known and perceived seriously by requesting physicians and the leaders. Such gaps can have many detrimental effects on the correct interpretation of the findings in the pathology specimens and consequently on patient safety. We hope that with official documentation of such inadequacies, this subject would be brought to the forefront of future quality improvement initiatives in pathology departments. Moreover, besides informing and educating the clinicians for diagnostic improvement and accuracy and handling the patient’s safety issues, one of the important factors is to use communication and tracking technologies such as hospital and laboratory information systems, and bar coding or RFID systems as potential solutions for specimen identification, tracking, and management in pathology. In the end, it would be informative if future studies document the impact of such information gaps on proper management of patient care and also the impact of effective interventions to tackle information inadequacies.

## Conflict of Interest

The authors declare no conflict of interest.
